# Identification of Stage-Specific Gene Expression Signatures in Response to Retinoic Acid during the Neural Differentiation of Mouse Embryonic Stem Cells

**DOI:** 10.3389/fgene.2012.00141

**Published:** 2012-08-07

**Authors:** Hiromi Akanuma, Xian-Yang Qin, Reiko Nagano, Tin-Tin Win-Shwe, Satoshi Imanishi, Hiroko Zaha, Jun Yoshinaga, Tomokazu Fukuda, Seiichiroh Ohsako, Hideko Sone

**Affiliations:** ^1^Health Risk Research Section, Center for Environmental Risk Research, National Institute for Environmental StudiesTsukuba, Ibaraki, Japan; ^2^Department of Environmental Studies, Graduate School of Frontier Science, The University of TokyoKashiwa, Chiba, Japan; ^3^Biological Impact Research Section, Center for Environmental Health Sciences, National Institute for Environmental StudiesTsukuba, Ibaraki, Japan; ^4^Department of Animal Production Science, Graduate School of Agricultural Science, Tohoku UniversitySendai, Miyagi, Japan; ^5^Center for Disease Biology and Integrative Medicine, Graduate School of Medicine, The University of TokyoTokyo, Japan

**Keywords:** mouse embryonic stem cells, neural differentiation, Bayesian network, retinoic acid, toxicity screening

## Abstract

We have previously established a protocol for the neural differentiation of mouse embryonic stem cells (mESCs) as an efficient tool to evaluate the neurodevelopmental toxicity of environmental chemicals. Here, we described a multivariate bioinformatic approach to identify the stage-specific gene sets associated with neural differentiation of mESCs. We exposed mESCs (B6G-2 cells) to 10^−8^ or 10^−7^ M of retinoic acid (RA) for 4 days during embryoid body formation and then performed morphological analysis on day of differentiation (DoD) 8 and 36, or genomic microarray analysis on DoD 0, 2, 8, and 36. Three gene sets, namely a literature-based gene set (set 1), an analysis-based gene set (set 2) using self-organizing map and principal component analysis, and an enrichment gene set (set 3), were selected by the combined use of knowledge from literatures and gene information selected from the microarray data. A gene network analysis for each gene set was then performed using Bayesian statistics to identify stage-specific gene expression signatures in response to RA during mESC neural differentiation. Our results showed that RA significantly increased the size of neurosphere, neuronal cells, and glial cells on DoD 36. In addition, the gene network analysis showed that glial fibrillary acidic protein, a neural marker, remarkably up-regulates the other genes in gene set 1 and 3, and *Gbx2*, a neural development marker, significantly up-regulates the other genes in gene set 2 on DoD 36 in the presence of RA. These findings suggest that our protocol for identification of developmental stage-specific gene expression and interaction is a useful method for the screening of environmental chemical toxicity during neurodevelopmental periods.

## Introduction

Humans are exposed to environmental chemicals on a daily basis; however, many effects of these chemicals on human health are unclear. Currently, assessment of developmental toxicity on children’s health is a large and rapidly growing research field. Children are not “little adults” and have special vulnerabilities to the toxic effects of environmental chemicals. For example, brain development during embryonic stages is an important period when microstructures are formed and axon guidance and synapse formation are induced by neuronal signaling (Lamoury et al., 2006; Ligon et al., 2006). These processes are regulated by stage-specific gene expression during embryonic development. Therefore, it is necessary to develop a more comprehensive and efficient system to identify the stage-specific gene expression signatures in embryonic development and to evaluate the toxicity of environmental chemicals on neural development.

Toxicity testing using embryonic stem cells (ESCs) has been developed as an efficient approach to assess the effect of environmental chemicals on neurodevelopment (Seiler et al., 2006). We have previously reported a mouse embryonic stem cell (mESC) neural differentiation protocol and showed that it could be used as an efficient tool to evaluate the toxic effects of environmental chemicals on neurodevelopment (Nagano et al., 2012). Furthermore, we have previously developed a method to quantitatively and statistically analyze microarray gene expression data using Bayesian networks with a log-linear functional relationship between genes (Toyoshiba et al., 2004, 2006). We proposed that advanced Bayesian network analysis is a necessary tool to understand the accurate linkage in the possible networks and the mechanism of the action of developmentally neurotoxic compounds.

During mammalian fetal development, the most active form of vitamin A, retinoic acid (RA) can pass through the umbilical cord to the fetus and induce axon formation and neural system development. ESCs express high levels of RA receptor (RAR)α in the undifferentiated stage, while RARβ begins to be expressed after embryoid body (EB) formation (Shiotsugu et al., 2004; Wilson and Maden, 2005; So et al., 2006). A series of RA concentrations were examined to detect neural cell identity during neuronal differentiation from mESC (Okada et al., 2004; Engberg et al., 2010). They reported that the 10^−8^ M of RA would be an optimum dose to induce cerebral and mesencephalic neurons and the 10^−7^ M of RA had capability to induce motor neurons (Kawasaki et al., 2000; Nishimura et al., 2003; Miyazaki et al., 2005).

Therefore, in the present study, we focused on identification of stage-specific gene expressions and analyzed their relationship network during mESC neurodevelopmental period after RA exposure at 10^−8^ and 10^−7^ M, using an advanced Bayesian network analysis.

## Materials and Methods

### Cell culture and differentiation

**Figure 1:**
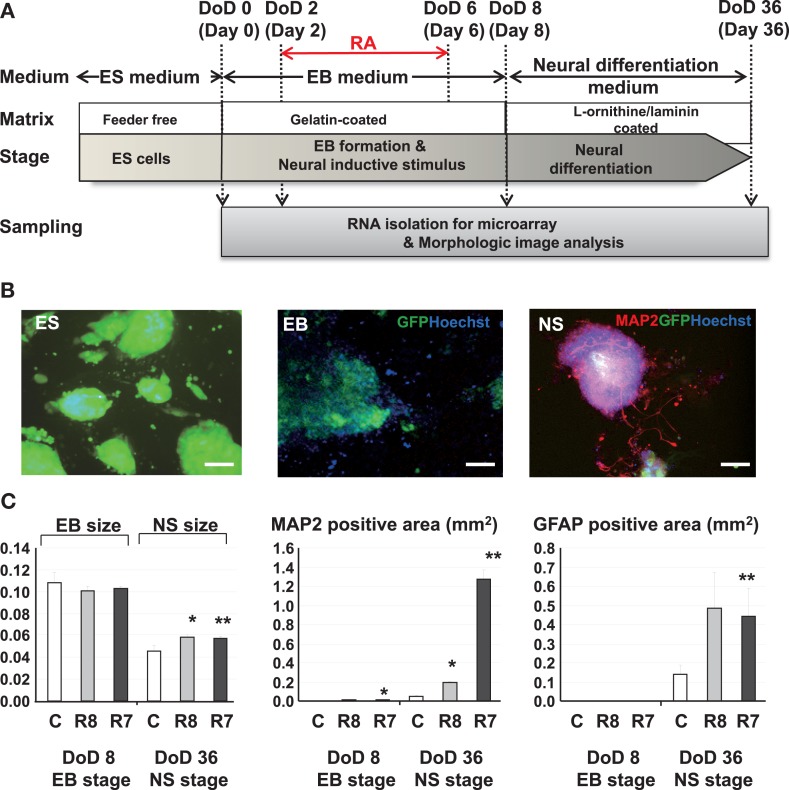
**Experimental protocols and morphological analysis of the effect of RA on the neural differentiation of mESCs**. **(A)** A Schematic diagram of cell cultures and chemical exposures. **(B)** Pictures of mESCs, EBs, and neurospheres (NS) with neurite growth derived from neurosphere in fluorescent fields. Green, blue, and red fluorescent images indicate GFP of cytoskeleton proteins, nuclei stained with Hoechst and neuronal cells and neurites stained with the anti *Map2* antibody. Scale bar is 100 μm. **(C)** Morphological analysis of neuronal cell lineages exposed to RAs. Comparison of the EB areas on DoD 8 and DoD 36 showed that the EB area decreased with neuronal cell development. In RA-treated EBs, the numbers of *Map2*-positive axons and *Gfap*-positive cells were increased compared with the control. Symbols of C, R8, and R7 indicate vehicle control, RA 10^−8^ M, and RA 10^−7^ M.

B6G-2 mESCs (RIKEN Cell Bank, Tsukuba, Ibaraki, Japan) were maintained in Dulbecco’s modified Eagle’s medium (Invitrogen, Carlsbad, CA, USA) supplemented with 15% knockout serum replacement (Invitrogen), 100 μM non-essential amino acids (Invitrogen), 100 μM 2-mercaptoethanol (Invitrogen), and 1000 U/ml leukemia inhibitory factor (LIF; Invitrogen) in gelatinized tissue culture dishes. On the first day of differentiation (DoD 0), cells were transferred in to 24 well plate in media without LIF and allowed to form EBs. The media was changed by every 2 days. RA was added during DoD 2–6 to induce neuronal differentiation. On DoD 8, EBs were transferred to l-ornithine/laminin-coated 24 well plates (BD Bio Coat, BD, Franklin Lakes, NJ, USA) and were cultured with neural medium from DoD 22 to DoD 36 to promote further neural differentiation (Figure 1A).

### Immunocytochemistry and morphological analysis

On DoD 8 and DoD 36, EBs, and their derivatives were fixed with 4% PFA in PBS for 15 min and then performed immunostaining with the conventional methods. Cells were incubated with primary antibodies overnight at 4°C at the following dilutions: anti-microtubule-associated protein 2 (*Map2*) antibody (Sigma-Aldrich, Poole, UK; 1:200) and mouse anti-glial fibrillary acidic protein (*Gfap*) monoclonal antibody (Chemicon International, Temecula, CA, USA; 1:200). Cells were rinsed with PBS and then incubated with Alexa-conjugated secondary antibodies (1:1000, Alexa Fluor 546, Invitrogen). Hoechst 33342 solution (Dojindo Laboratories, Kumamoto, Japan) was used for counter-staining. Immunofluorescence images were acquired with six biological replicates per condition using an IN Cell Analyzer 1000 (GE Healthcare, Buckinghamshire, UK) and analyzed using IN Cell Developer Tool Box 1.7 (GE Healthcare). All morphological analysis experiments were performed in triplicate to test the reproducibility of the results. Statistical analysis was performed using two-tailed Student’s *t*-test. Relationships were considered statistically significant with *p* < 0.05.

### DNA microarray analysis

Total RNA was isolated on DoD 0, 2, 8, and 36 with six biological replicates. And then, single mixed RNA sample per condition was applied to Illumina MouseWG-6v1.0 expression BeadChips covering 46,643 transcripts including 26,766 annotated coding transcripts 2, according to the manufacturer’s instructions (Illumina, San Diego, CA, USA). The arrays were scanned in accordance with the manufacturer’s directions. Raw expression values of each gene were normalized with median centered by GeneSpring GX10.02 software (Agilent Technologies, Palo Alto, CA, USA). Normalized data were deposited in the National Center for Biotechnology Information Gene Expression Omnibus^1^ (accession no. GSE37602).

### Selection of gene sets

To capture gene expression signatures of stage-specific changes during neural differentiation of mESCs, we performed three approaches to determine gene sets for Bayesian network analysis. Marker genes, which are commonly used to analyze pluripotency and development of neural cells, were selected as the literature-based gene set (set 1) by review of the published literature. The analysis-based gene set (set 2) was selected by the combined use of the knowledge-based database and the following classification methods. Candidate genes involved in axon guidance maps, the nerve growth factor (NGF) pathway, and RA signaling were preliminarily selected from the Kyoto Encyclopedia of Genes and Genomes (KEGG) pathway database^2^ and then genes with specific expression patterns were identified using SOM and PCA. Finally, the enrichment gene set (set 3) was selected by clustering expression values of candidate genes contained in the Neurogenesis and Neural Stem Cell PCR Array (SABiosciences, Valencia, CA, USA) using SOM and PCA. SOM and PCA were performed using GeneSpring GX10.02 software (Agilent Technology). Briefly, SOM clustering was done by conditions in which similarity measure: euclidean, maximum number of iterations: 50, numbers of grid rows and columns were 2 × 4. Then each eight clusters of SOM were analyzed by PCA with four components of eigenvalues (component 1 was more than 40% and component 2 was 10%). To develop set 2 and 3, we collected genes with maximum and minimum values in the PCA component 1 from each SOM cluster.

### Gene interaction network analysis

We used a modified gene interaction network (GIN) based on our previous studies (Yamanaka et al., 2004; Toyoshiba et al., 2006; Nagano et al., 2012). The GIN was quantified to calculate the posterior probability distribution for the strength of the linkages based on gene expression and chemical exposure dose datasets. Briefly, a GIN consists of a collection of P nodes, denoted *G*_1_, *G*_2_, …, *G*_P_, with observed values *n*_1_, *n*_2_, … *n*_p_. β*ij* (*i*, *j* = 1, 2, …, P) are parameters in the log-linear function form describing the linkage from node *i* to node *j*. Mathematically, this is written as

$$E[\log(G_{j})]=\sum_{i=1,\neq j}^{P}I_{ij}{\text{β}}_{ij}\log(n_{i})$$

where *E*[log(*G_j_*)] represents the expectation for the natural logarithm of *G_j_*, and *I_ij_*(*i, j* = 1, 2, …, P) is an indicator function that equals 1 if node *G_i_* has a link to node *G_j_*, otherwise it equals 0. If a node has a regulatory effect on node *G_i_*, then that node is referred to as a “Parent of node *G_i_*,” and we refer to it as belonging to the set Pa (*G_i_*). The prior distribution for *I_ij_* was assumed to be a Bernoulli distribution with success probability *p_ij_* when *Iij* = 1. In the uninformative case, *p_ij_* could be set to 0.5 and if there is some expectation that *I_ij_* is not equal to zero, the prior probability could be set higher. The posterior distributions for the linkages were derived using Gibbs sampling. The network was used to evaluate the ability of the algorithm to have a higher posterior probability (*p*-value). Transition matrices were generated at *p* > 0.5.

## Results

### Effects of RA on neural differentiation

Exposure to RA at different concentrations during EB formation induced neuronal and glial cell lineages from mESCs (Figure 1A). Morphological analysis with immunofluorescent staining showed that RA significantly increased the size of neurosphere, neuronal cells, and glial cells at DoD 36 (Figures 1B,C).

### Gene set selection for gene network analysis

**Figure 2:**
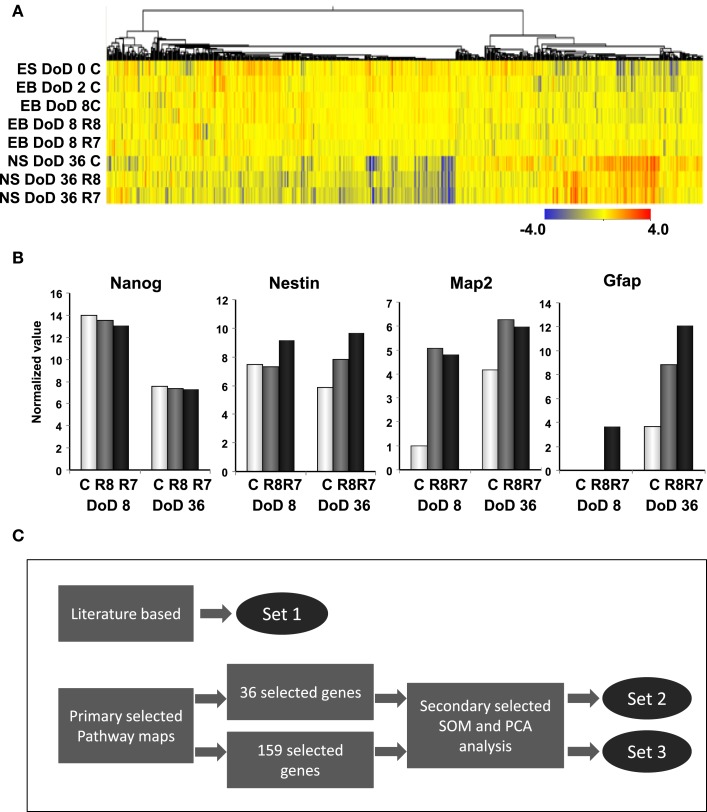
**Gene expression analysis by DNA microarray and gene selection strategies for the Bayesian network analysis of differentiation of neuronal cells derived from mESCs**. **(A)** Heat map of hierarchical clustering generated from DNA microarray data. Color-coding in the heat map is that blue from red indicates – 4.0 from 4.0 log2 normalized intensity value by ES values, indicating that red is for up regulation and blue is for down regulation. **(B)** Gene expression of pluripotency and differentiation markers in mESCs, EB, and NS measured in DNA microarray. Symbols of C, R8, and R7 indicate vehicle control, RA 10^−8^ M, and RA 10^−7^ M. **(C)** Stage-specific gene expression signatures in response to RA during the neural differentiation of mESCs were identified as follows: set 1 was a set of genes selected from the literature; set 2 was selected by SOM and PCA after selecting 36 genes from pathway maps; set 3 was selected by SOM and PCA after selecting 159 genes from pathway maps. Expression values of microarray data corresponding to genes in these three sets were used for the Bayesian network analysis.

To investigate transcriptomic changes as a result of neuronal differentiations and influences of RAs, a cDNA microarray was used to compare expression levels with and without the RA treatments in EB formations and neurosphere developments by hierarchical clustering methods (Figure 2A). From 22,188 transcripts presented from eight microarrays, 1,157 transcripts with expression differences greater than 2.0-fold in at least 1 microarray were selected for further analysis. From the microarray analysis, *Nanog* as a marker of undifferentiated ESCs and, *Nestin*, *Map2*, and *Gfap* as markers of neural cells were differentially expressed by RA treatments at differential doses during the neural differentiation of mESCs, suggesting that our protocol could detect the effects of RA on neuronal differentiation (Figure 2B). A high level of *Nanog* expression on DoD 8 was decreased in a dose-dependent fashion following RA treatments, but not on DoD 36. *Nestin* expression was increased by the 10^−7^ M RA treatment on DoD 8 and DoD 36. *Map2* and *Gfap* expressions were also increased by RA treatments on DoD 8 and DoD 36 (Figure 2B).

Three gene sets were selected for the Bayesian network analysis by our strategies as shown in Figure 2C. Selected gene sets are listed in Table 1. Concretely, set 1 was selected by the review of published articles and included *Nanog* (Mitsui et al., 2003; Loh et al., 2006), *Pou5f1* (Okazawa et al., 1991; Catena et al., 2004; Akamatsu et al., 2009), *Zfl42* (Shi et al., 2006; Scotland et al., 2009), *Fgfr1* (Jukkola et al., 2006; Yang et al., 2008; Lee et al., 2009), *Sox2* (Tomioka et al., 2002; Graham et al., 2003; Tanaka et al., 2004; Jin et al., 2009), and *Oligo2* (Ahn et al., 2008). *RARs* were also added to set 1 to assess the effects of RA. Set 2 was selected by the combined use of the KEGG database and SOM and PCA classification methods. Firstly, a list of 36 candidate genes was compiled according to axon guidance, NGF pathway, and RA signaling of KEGG database. It is known that NGF can induce neuronal differentiation of mESCs (Schuldiner et al., 2001) while RA can induce the expression of the NGF receptor (p75) during the neuronal differentiation of PC12 cells (Cosgaya et al., 1996). Therefore, genes in the NGF pathway were selected as indicators to assess the effects of RA on the neural differentiation of mESCs. The 36 candidate genes were then classified in to 17 classes by SOM, and representative genes were selected from each class by PCA. Finally, 16 genes were selected for set 3 by SOM and PCA clustering from 159 candidate genes contained in the Neurogenesis and Neural Stem Cell PCR Array (SABiosciences). Furthermore, specific markers for astrocytes (*Gfap*), mature neurons (*Map2*), neuronal stem cells (*Nestin*), and young neurons (*Tuj1*) were added to sets 2 and 3 to assess the stage of neuronal differentiation.

**Table 1:** **Lists of gene sets for identifying gene networks**.

Category	Set1	Set2	Set3
Pathway signaling		Map2k1	Adora2a
		Mapk1	Drd5
		Mapk3	Fgf13
		Pla2g6	Gnao1
		Rps6ka1	Notch2
		Shc1	Tnr
Transcription/chromatin regulation	RARa	Atbf1	Ascl1
	RARb	Cdyl	Gusb
	RARg	Rhog	Mef2c
	Nanog	Rif1	Pax5
	Pou5f1	Sall1	Pou3f3
	Zfp42	Smarcad1	
Neural development	Fgfr1	Fos	Bdnf
	Olig2	Gbx2	Gdnf
	Sox2	Hras1	Nrp2
		Raf1	Slit2
		Sox2	Ywhah
Neural marker	Gfap	Gfap	Gfap
	Map2	Map2	Map2
	Nestin	Nestin	Nestin
	Tuj1	Tuji1	Tuji1

### Gene interaction analysis

**Figure 3:**
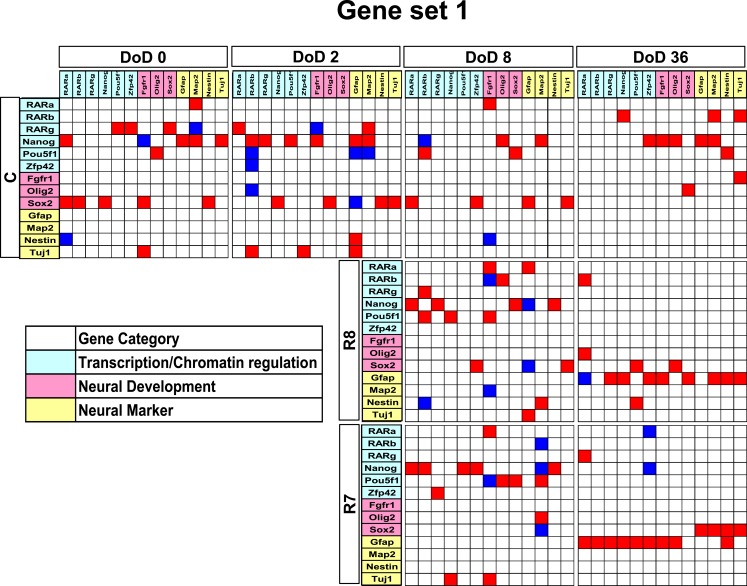
**Matrices in gene interaction networks of the literature-based gene set (set 1) in mESCs, EB, and NP of neuronal differentiation**. Gene Symbols, *RARa*, *RARb*, *RARg*, *Nanog*, *Pou5f1*, *Zfp42*, *Fgfr1*, *Olig2*, *Sox2*, *Gfap*, *Map2*, *Nestin*, *Tuj1*, line up in order of up to down in the *y* axis and left to right in the *x* axis. Red indicates upregulated genes and blue indicates downregulated genes. Red indicates that genes from the *y* axis upregulated genes from the *x* axis. Blue indicates that genes from the *y* axis downregulated genes from the *x* axis. Symbols of C, R8, and R7 indicate vehicle control, RA 10^−8^ M, and RA 10^−7^ M.

**Figure 4:**
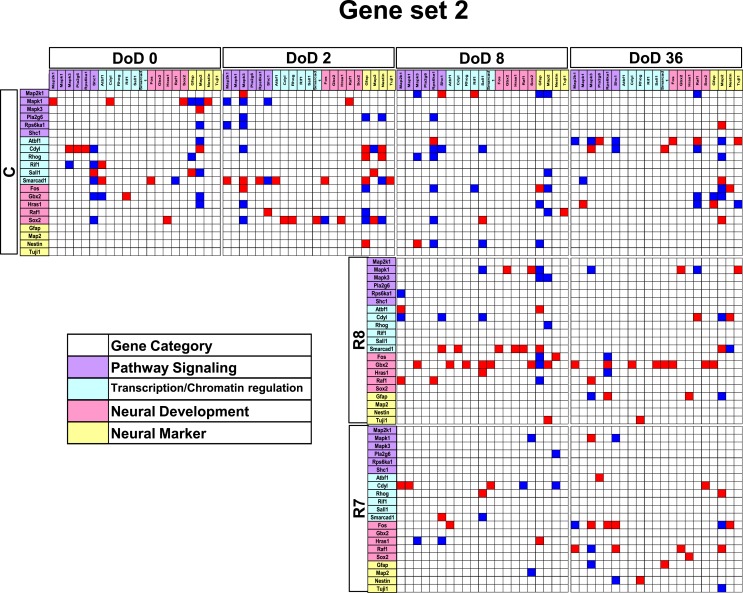
**Matrices in gene interaction networks of the analysis-based gene set (set 2) in mESCs, EB, and NP of neuronal differentiation**. Gene Symbols, *Map2k1*, *Mapk1*, *Mapk3*, *Pla2g6*, *Rps6ka1*, *Shc1*, *Atbf1*, *Cdv1*, *Rhog*, *Rif1*, *Sall1*, *Smarcad1*, *Fos*, *Gbx2*, *Hras1*, *Raf1*, *Sox2*, *Gfap*, *Map2*, *Nestin*, *Tuji1* line up in order of up to down in the *y* axis and left to right in the *x* axis. Red indicates upregulated genes and blue indicates down regulated genes. Red indicates that genes from the *y* axis up regulated genes from the *x* axis. Blue indicates that genes from the *y* axis downregulated genes from the *x* axis. Symbols of C, R8, and R7 indicate vehicle control, RA 10^−8^ M, and RA 10^−7^ M.

**Figure 5:**
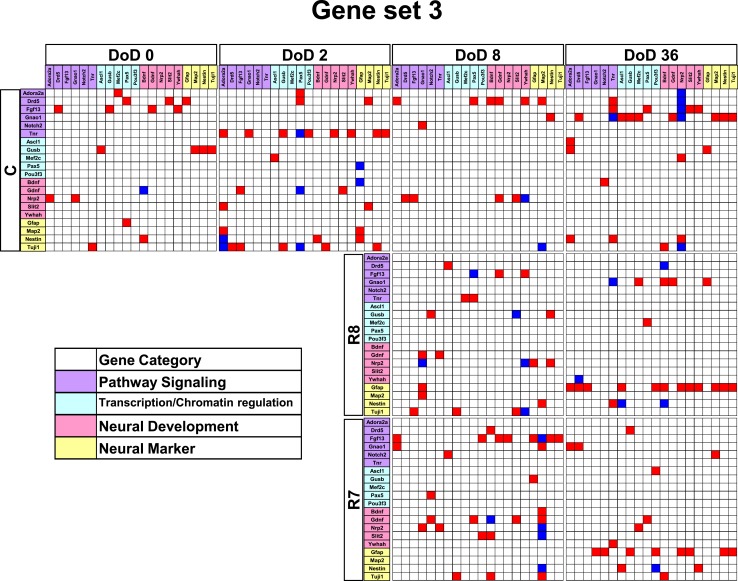
**Matrices in gene interaction networks of the enrichment gene set (set 3) in mESCs, EB, and NP of neuronal differentiation**. Gene Symbols, *Adora2a*, *Drd5*, *Fgf13*, *Gnao1*, *Notch2*, *Tnr*, *Ascl1*, *Gusb*, *Mef2c*, *Pax5*, *Pou3f3*, *Bdnf*, *Gdnf*, *Nrp2*, *Slit2*, *Ywhah*, *Gfap*, *Map2*, *Nestin*, *Tuji1* line up in order of up to down in the *y* axis and left to right in the *x* axis. Red indicates up regulated genes and blue indicates down regulated genes. Red indicates that genes from the *y* axis up regulated genes from the *x* axis. Blue indicates that genes from the *y* axis down regulated genes from the *x* axis. Symbols of C, R8, and R7 indicate vehicle control, RA 10^−8^ M, and RA 10^−7^ M.

**Figure A1:**
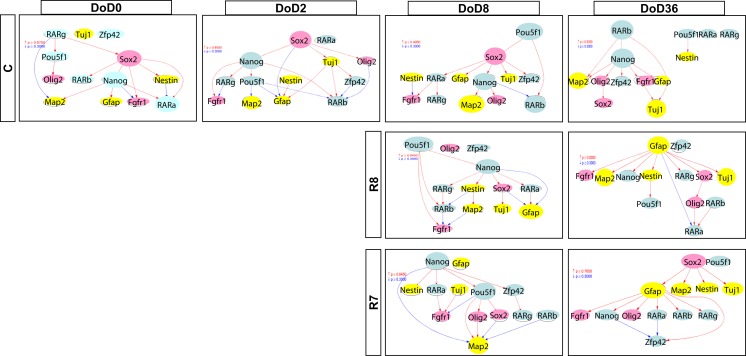
**Gene interaction networks of the literature-based gene set (set 1) in mESCs, EB, and NP of neuronal differentiation**. The optimized network was generated at *p* > 0.300. Red indicates that parent genes up-regulate children genes. Blue indicates that parent genes down-regulate children genes.

**Figure A3:**
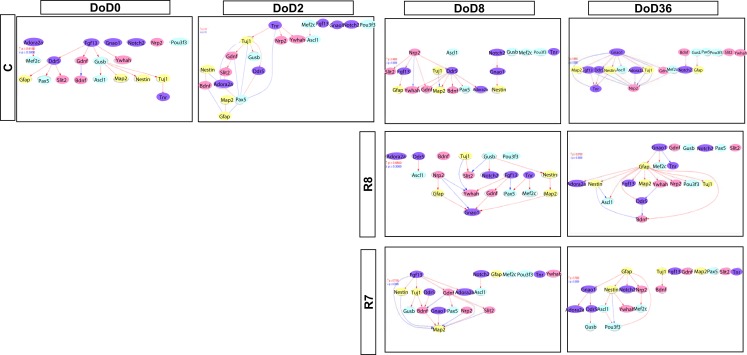
**Gene interaction networks of the enrichment gene set (set 3) in mESCs, EB, and NP of neuronal differentiation**. The optimized network was generated at *p* > 0.300. Red indicates that parent genes up-regulate children genes. Blue indicates that parent genes down-regulate children genes.

Matrices transferred from gene interaction analysis for set 1, set 2, and set 3 are shown in Figures 3–5, respectively (see Figures A1–A3 in Appendix as references and Tables S1–S6 in Supplementary Material for input data and output raw-results). In the control group of set 1, *Nanog*, and Sox2 (Figure 3) which control ESC pluripotency, regulate many other genes on DoD 0, 2, and 8. On DoD 36, Sox2 does not regulate any gene. In RA-treated groups of set 1, linkages of RARs in the matrix indicated that these genes might play principal roles in the regulation of expression of other genes. Briefly, the effect of RA was observed on DoD 8, in which RA 10^−8^ or 10^−7^ M aggravated *Nanog* and *Pou5f1*. On DoD 36, the matrix was more strongly influenced by RA, in which the neural marker genes such as *Gfap* and *Map2* up-regulated the other genes, indicating that RA enhances neural differentiation (Figure 3).

Gene interaction matrix analysis for set 2 is shown in Figure 4. In the mESC matrix, linkages between genes were concentrated to categories of pathway signaling and neural development, which is similar with those in set 1 on DoD 8 and DoD 36. It is noteworthy that Gbx2 as a neuronal development marker strongly up-regulated *Mapk3*, *Atbf1*, *Rhog*, *Sall1*, *Smarcad*, *Sox2*, and *Map2* on DoD 8 as well as DoD 36 in RA 10^−8^ M matrices. RA-treated matrices showed that linkages shifted to the right side of the matrix with increasing RA concentrations. Finally, linkages on DoD 36 were concentrated to categories of neural development and neural markers.

Gene interaction network analysis for set 3 is shown in Figure 5. In the mESC matrix, linkages between genes were concentrated to transcription/chromatin regulation and pathway signaling categories. Linkages between genes in the control matrix on DoD 8 were concentrated to pathway signaling and neural development categories. In the RA-treated matrices, linkages between genes moved to the neural marker category from the neural development category in a dose-dependent manner. Most of the linkages between genes in the RA-treated matrices on DoD 36 were concentrated to pathway signaling and neural marker categories, suggesting that *Gfap* mainly regulates neuronal differentiation.

## Discussion

In the present study, a prediction model for the neural differentiation of mESCs was established and stage-specific gene expression signatures in response to RA were identified using Bayesian network analysis. Our present findings showed that RA significantly increased the size of neurosphere, neuronal cells, and glial cells on DoD 36. In addition, neural marker *Gfap* remarkably up-regulated the other genes in gene set 1 and 3, and neural development marker *Gbx2* significantly up-regulated the other genes in gene set 2 on DoD 36 in the presence of RA. These findings suggest that our protocol for identification of developmental stage-specific gene expression and interaction is a useful method for the screening of environmental chemical toxicity during neurodevelopmental periods.

RA is known as a severe teratogen and causes central nervous system malformations. However, *in vivo* study indicated that high dose (70 mg/kg body weight; b.w.) of RA could induce teratogenic effects during gestational day 7–9 in Swiss mice (Veiga Quemelo et al., 2007). In addition, it was reported that the physiological dose that cannot affect RAR level was 1 mg/kg b.w. and minimally teratogenic dose was 10 mg/kg b.w. and completely teratogenic dose was 100 mg/kg b.w. in gestational day 9 of mouse (Harnisha et al., 1990). In the present study, we selected the dose of RA as 10^−8^ and 10^−7^ M because endogenous levels of RA-induced neural differentiation in the early embryo are approximately 1–10 nM (Maden et al., 1998; Mic et al., 2003). Therefore, we considered to use 10^−8^ M as a low dose and 10^−7^ M as a high dose to examine the effect of RA on stage-specific gene expression signature in mESCs.

We have also successfully designed a mESC neural differentiation protocol to evaluate the effect of RA on the neural differentiation of mESCs. Morphological analysis using a high-content image analyzer was able to acquire varying differences of differentiation from mESCs to neural cells by the RA treatment. For instance, neuronal or glial differentiation from neuronal ESCs was delayed in control cells without induction by RA, which was further confirmed by the lower expression levels of *Map2* and *Gfap* detected on DoD 36. RA treatments promoted the loss of pluripotency and differentiation into neural ESCs up to DoD 36 in the present study (Figures 1B,C), suggesting that the maturation of *Map2*-positive neurons and *Gfap*-positive astrocytes were accelerated by RA treatment. Our results are consistent with a study showing that RA and LIF enhance the induction of *Gfap*-positive astrocytes from mice neural progenitor cells via epigenetic modifications (Asano et al., 2009).

In restricted sample size analysis like the present study, simulations using Bayesian network analysis have been suggested to be a very effective method (Toyoshiba et al., 2006). Our present study provided a new experimental evidence that Bayesian network analysis was effective to identify the functions of the well-known neural development regulators, such as *Gfap* and *SOX2* (Figures 3–5), in response to RA during the neural differentiation of mESCs and suggested its further application to predict developmental neurotoxicity of environmental chemicals. However, in the simulation analysis, one major problem is to select genetic markers related with a trait of interest. To perform accurate simulation, it is undesirable to select genes with similar expression patterns. Similar variables could significantly affect the analysis results and potentially lead to biased results. Hence, the selection of genes with distinct expression patterns, which can represent each stage of mESC neural differentiation, seems to be important in the outcome of the GIN analysis. In this study, we selected gene sets for GIN analysis by the combined use of two classification methods, SOM and PCA. SOM is a powerful data mining method, whose algorithm is an unsupervised competitive learning neural network and it maps high-dimensional data into a simple low-dimensional display (Kohonen, 1990; Zhang et al., 2008). Therefore, SOM is able to classify the temporal expression data for each gene. After classification by SOM based on gene expression patterns, the representative genes were further selected from each class by PCA. PCA is a standard technique of pattern recognition and has been widely used as a tool in exploratory data analysis and for making predictive models in many biological systems (Aiba et al., 2006; van Dartel et al., 2010; Qin et al., 2011). In this study, the genes selected by SOM and PCA were shown to have adequate simulation parameters to evaluate the effects of RA on the neural differentiation of mESCs.

Finally, our prediction model, employing Bayesian network analysis, showed that it is possible to capture genetic correlations between genes and to identify slight variations for different conditions. We performed the same prediction model for three gene sets of different genetic constitution. Our study indicated that the GIN was able to capture features of each developmental stage during the neural differentiation of mESCs. RA treatment could change the network structure in a dose-dependent manner. In addition, among the three gene sets, set 3 was the best according to the morphological results. We found that the *Gfap* gene was linked with other genes in the RA 10^−7^ M matrix in the GIN analysis, while the number of *Gfap*-positive cells was markedly increased by RA 10^−7^ M treatment on DoD 36 in the morphological analysis. This suggested that the approach used in this study, of the independent selection of gene sets using SOM or PCA, was efficient. This Bayesian model might also be useful to investigate the developmental toxicity of environmental chemicals other than RA.

In summary, to find the optimized GIN that integrated chemical effects, we created three different gene sets and then performed GIN analysis using Bayesian network algorithms to capture the stage-specific gene expression signatures in response to RA treatment during the neural differentiation of mESCs. “*Toxicity Testing in the Twenty First Century – A vision and a strategy*” issued by the US Nuclear Regulatory Commission indicated that the most important issue for toxicity testing is how to connect the extensive body of toxicity information to high-throughput screening to perform chemical risk assessment (Thomas et al., 2007; Davis et al., 2008; Ellinger-Ziegelbauer et al., 2009; Hubal, 2009). Here, we described a novel approach to identify stage-specific gene expression in embryonic development and suggested its application to evaluate the neural developmental toxicity of environmental chemicals in future studies.

## Conflict of Interest Statement

The authors declare that the research was conducted in the absence of any commercial or financial relationships that could be construed as a potential conflict of interest.

## Supplementary Material

The Supplementary Material for this article can be found online at: http://www.frontiersin.org/Toxicogenomics_/10.3389/fgene.2012.00141/abstract

Supplementary Table S1 **Input data of gene expression values for set 1**. Values are normalized expression values in beads of each gene with approximate 30 replicates.

Supplementary Table S2 **Input data of gene expression values for set 2**. Values are normalized expression values in beads of each gene with approximate 30 replicates.

Supplementary Table S3 **Input data of gene expression values for set 3**. Values are normalized expression values in beads of each gene with approximate 30 replicates.

Supplementary Table S4 **Output results of the Bayesian network analysis with gene expression values for set 1**. Values are posterior probabilities.

Supplementary Table S5 **Output results of the Bayesian network analysis with gene expression values for set 2**. Values are posterior probabilities.

Supplementary Table S6 **Output results of the Bayesian network analysis with gene expression values for set 3**. Values are posterior probabilities.

## Figures and Tables

**Figure A2:**
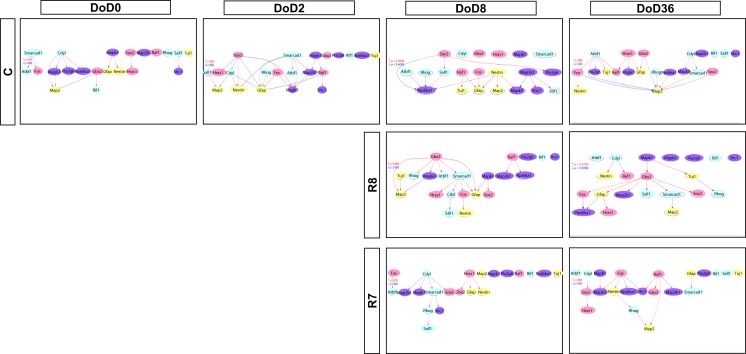
**Gene interaction networks of the analysis-based gene set (set 2) in mESCs, EB, and NP of neuronal differentiation**. The optimized network was generated at *p* > 0.300. Red indicates that parent genes up-regulate children genes. Blue indicates that parent genes down-regulate children genes.

## Footnotes

^1^www.ncbi.nlm.nih.gov/geo

^2^ http://www.genome.jp/kegg/pathway.html
